# Gender- and Age-Related Changes in Trunk Muscle Composition Using Chemical Shift Encoding-Based Water–Fat MRI

**DOI:** 10.3390/nu10121972

**Published:** 2018-12-13

**Authors:** Egon Burian, Jan Syväri, Christina Holzapfel, Theresa Drabsch, Jan S. Kirschke, Ernst J. Rummeny, Claus Zimmer, Hans Hauner, Dimitrios C. Karampinos, Thomas Baum, Daniela Franz

**Affiliations:** 1 Department of Diagnostic and Interventional Neuroradiology, Klinikum rechts der Isar, Technical University of Munich, Ismaninger Str. 22, 81675 Munich, Germany; jan.kirschke@tum.de (J.S.K.); claus.zimmer@tum.de (C.Z.); thomas.baum@tum.de (T.B.); 2 Department of Diagnostic and Interventional Radiology, Klinikum rechts der Isar, Technical University of Munich, Ismaninger Str. 22, 81675 Munich, Germany; jan.syvaeri@tum.de (J.S.); ernst.rummeny@tum.de (E.J.R.); dimitrios.karampinos@tum.de (D.C.K.); daniela.franz@tum.de (D.F.); 3 Institute of Nutritional Medicine, Klinikum rechts der Isar, Technical University of Munich, Georg-Brauchle-Ring 62, 80992 Munich, Germany; christina.holzapfel@tum.de (C.H.); theresa.drabsch@tum.de (T.D.); hans.hauner@tum.de (H.H.)

**Keywords:** skeletal muscle, magnetic resonance imaging, muscular fat deposition, obesity, BMI

## Abstract

Ageing, sarcopenia, and malnutrition are associated with quantitative and qualitative changes of body composition. There are several imaging modalities, including magnetic resonance imaging (MRI), for the assessment of trunk muscle tissue composition. In this study, we investigated the gender- and age-related changes in trunk muscle composition using chemical shift encoding-based water–fat MRI. A total of 79 healthy volunteers (26 men: 38.9 ± 10.4 years; 53 women: 39.5 ± 15.0 years) underwent 3T axial MRI using a six-echo multi-echo 3D spoiled gradient echo sequence, allowing for the calculation of the proton density fat fraction (PDFF) in the trunk muscles. PDFF of the abdominal, psoas, and erector spinae muscles were determined. We detected significant positive correlations for abdominal muscle PDFF with age (*r* = 0.638, *p* = 0.0001) in men, and for abdominal muscle PDFF (*r* = 0.709, *p* = 0.0001) and erector spinae muscle PDFF (*r* = 0.674, *p* = 0.0001) with age in women. After adjustment for body mass index (BMI), only the correlation of age and abdominal muscle PDFF in women remained significant (*r* = 0.631, *p* = 0.0001). The findings of this study suggest that an increasing fat deposition in muscle is driven primarily by age, rather than BMI, in women. These results further support that PDFF can be considered a valid imaging biomarker of trunk muscle composition.

## 1. Introduction

During the last decade, several environmental and lifestyle changes emerged, favoring a positive energy balance and weight gain. An ageing population, increasing high caloric per capita food intake, a decreasing time spent with physical exercise, and prevailing sedentary activities cause a positive energy balance [1]. These changes have a variety of pathophysiological, psychological, and socioeconomic consequences [2,3,4,5,6]. A dysregulation of energy homeostasis may produce adiposity, characterized by an excess accretion of lipids in adipose tissues [7]. Subcutaneous and visceral adipose tissue contain most of the stored lipids [8]. However, a proportionately elevated level of fat deposition in bone marrow and muscular tissue was extensively described in the past, and is likewise associated with a higher risk of metabolic, cardiovascular, and inflammatory diseases [9,10,11,12,13,14]. In addition to the ectopic fat deposition in muscle, senescence-related alterations in skeletal muscle, like fatty replacement of paraspinal muscles, are associated with adverse outcomes, such as vertebral fractures and even death [15,16].

Besides anthropometry and bioimpedance analysis, there are several imaging modalities for determining body composition and fat distribution. The use of dual-energy X-ray absorptiometry (DXA) provides estimates of bone mineral content, lean soft tissue, and adipose tissue mass for the whole body [17]. With computed tomography (CT), a highly accurate quantification of whole body and regional muscle mass and composition is possible, thereby detecting subtle volumetric changes over time [18]. Magnetic resonance imaging (MRI), as a method free of ionizing radiation with high soft tissue resolution, has gained importance during the last years. Different techniques like single-voxel proton magnetic resonance spectroscopy (MRS) and chemical shift encoding-based water–fat MRI have been used until today, allowing for calculation of surrogate parameters like the proton density fat fraction (PDFF), cross-sectional area (CSA), and even for the identification of the chemical structure of fatty acids and their magnitude [19,20,21]. As MRS in the abdominal region is particularly prone to breathing artifacts, chemical shift encoding-based water–fat MRI is the better option for fast and robust trunk muscle composition analysis. PDFF mapping, based on a multi-echo gradient echo acquisition, has been shown to allow spatially resolved fat quantification in multiple organs, and liver PDFF is emerging as an important metabolic phenotyping parameter [22]. In the past, it has been shown that extracting PDFF from chemical shift encoding-based water–fat MRI is a reliable approach to obtain quantitative information about the water–fat composition of different body compartments, like vertebral bone marrow or paraspinal musculature, with good concordance between PDFF measurements and histology or MRS [20,23]. Thus, MRI allows for calculation of fatty infiltration parameters and the visualization of structural changes in muscular architecture beyond sole body mass index (BMI) calculation with impact on functionality [24]. However, the impact of age and BMI on the structural changes of trunk musculature composition and ectopic fat accumulation using PDFF measurements has not been investigated. Given the close spatial and functional interactions of the trunk musculature and the vertebral column, elucidating the connection of ageing, BMI, and changing PDFF is expected to offer novel insights into muscle pathophysiology.

Even with skeletal muscle being the largest body compartment in adults, besides excessive adipose tissue in the presence of obesity, we are still at the beginning of understanding the functional consequences of changes in muscle mass [25,26]. The aim of the present analysis was to improve our knowledge on the measurement of fatty infiltration of trunk muscle groups, taking into account gender and age, using advanced quantitative MRI techniques. Therefore, the purpose of the present study was to investigate the gender- and age-related changes in PDFF and CSA of the abdominal, psoas, and erector spinae muscles of healthy adults, using chemical shift encoding-based water–fat MRI.

## 2. Materials and Methods

### 2.1. Subjects

Volunteers (*n* = 111; 76 women and 34 men) were recruited at the Institute for Nutritional Medicine, Klinikum rechts der Isar, Technical University of Munich, from a large study aimed at investigating determinants of basal metabolic rate [27]. The study protocol and all procedures were approved by the ethical committee of the Faculty of Medicine of the Technical University of Munich, Germany. Subjects were screened for eligibility, and included if age was equal to or greater than 18 years. Subjects who appeared healthy, according to self-report, showed no history of severe diseases or surgery within the last three months, and did not have acute physical impairment were eligible. Pregnant and breastfeeding women, as well as subjects with standard contraindications for MRI examinations, were excluded. All subjects gave written informed consent before participation in the study. Of the 111 volunteers, 79 subjects (26 men: 38.85 ± 10.38 years; 53 women: 39.51 ± 15.03 years) received an additional 3D spoiled gradient echo sequence allowing for the calculation of PDFF in the trunk muscles.

### 2.2. MR Imaging (T2 mDixon Quant)

Subjects underwent 3 T MRI (Ingenia, Philips Healthcare, Best, Netherlands). An axial six-echo multi-echo 3D spoiled gradient echo sequence was used for chemical shift-encoding based water–fat separation at the abdomen using the anterior and posterior coil arrays. The sequence acquired the six echoes in a single TR using non-flyback (bipolar) read-out gradients, and covered the entire abdomen in two axial stacks (starting from the top of the liver and covering a feet/head distance of 300 mm) with the following imaging parameters per stack: TR/TE1/ΔTE = 7.8/1.35/1.1 ms, FOV = 300 × 400 × 150 mm^3^, foldover suppression in both L/R directions with 50 mm, acquisition matrix size = 152 × 133, acquisition voxel size = 2 × 3 × 6 mm^3^, SENSE with reduction factor = 2.2 × 1.2, receiver bandwidth = 1678 Hz/pixel, frequency direction = A/P (to minimize breathing artifacts), 1 average, acquired in a 15 s breath-hold. A flip angle of 3° was used to minimize T1-bias effects.

### 2.3. PDFF Mapping

The gradient echo imaging data were processed online using the fat quantification routine of the MR vendor (mDixon, Philips Healthcare, Best, Netherlands). The routine procedure performs a phase error correction and a complex-based water–fat decomposition using a pre-calibrated seven-peak fat spectrum as well as a single T_2_* correction to model the signal variation with echo time. The imaging-based PDFF map was then computed as the ratio of the fat signal over the sum of fat and water signals.

### 2.4. PDFF and CSA Calculation

**Figure 1:**
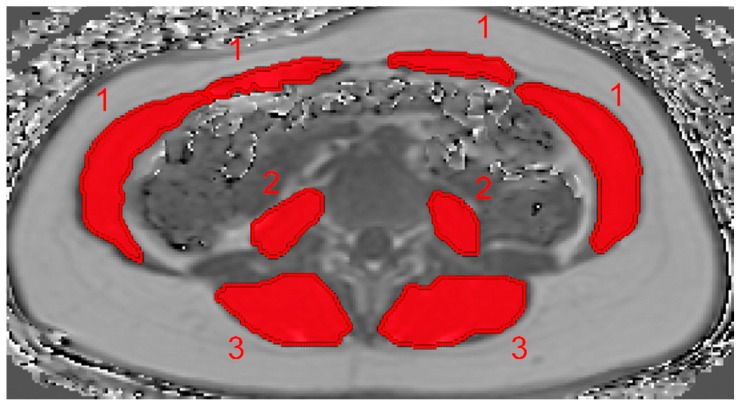
Representative segmentation of the abdominal muscles, psoas muscle, and erector spinae muscle in the proton density fat fraction (PDFF) map of a 40-year-old woman. The rectus abdominis muscle and internal oblique muscle (1), psoas muscle (2), and erector spinae (3) are segmented at the level of L4.

The abdominal muscles, psoas muscle, and erector spinae were included in the analysis and manually segmented in the PDFF maps by a radiologist in a single slice at midvertebral L4 level (Figure 1) bilaterally. Segmentation was performed by using the free open-source software Medical Imaging Interaction Toolkit (MITK, developed by the Division of Medical and Biological Informatics, German Cancer Research Center, Heidelberg, Germany). PDFF and CSA values of each ROI were recorded, and the average value of both sides was calculated.

### 2.5. BMI Calculation

Height was measured without shoes in a standing position using a stadiometer (Seca, Hamburg, Germany), and reported to the nearest 0.1 cm. Weight on the day of the MRI scan was self-reported. BMI was calculated as the quotient of weight and height squared (kg/m^2^).

### 2.6. Statistical Analysis

The statistical analyses were performed with SPSS (SPSS Inc., Chicago, IL, USA). All tests were performed using a two-sided 0.05 level of significance. The presented figures were generated using GraphPad Prism Version 7 (Version 7.0, Graphpad Software Inc., La Jolla, CA, USA).

The Kolmogorov–Smirnov test indicated no normally distributed data for the majority of parameters. Mean and standard deviation (SD) of PDFF and CSA in the three muscle compartments of 26 men and 53 women were extracted, calculated, and compared using Mann–Whitney tests. Correlations of PDFF and CSA with age and BMI were analyzed using Spearman’s rho correlation coefficients. BMI and age were defined as potential confounding factors influencing trunk muscle PDFF, and partial correlations were calculated to adjust for BMI and age, respectively.

## 3. Results

### 3.1. Study Population

There was no statistically significant difference between men and women in terms of age (men: 38.85 ± 10.38 years, range: 26–61 years; women: 39.51 ± 15.03 years, range: 21–77 years; *p* = 0.665) and BMI (men: 26.37 ± 5.30 kg/m^2^, range: 19.95–44.51 kg/m^2^; women: 25.67 ± 5.46 kg/m^2^, range: 19.26–43.51 kg/m^2^; *p* = 0.455) (Table 1). Eight male and 21 female subjects were under 30 years, 12 male and 18 female subjects were between 31 and 49 years, and 6 male and 14 female subjects were older than 49 years.

### 3.2. PDFF and CSA Measurements

**Figure 2:**
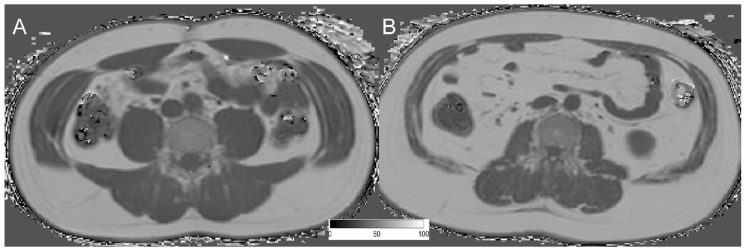
Representative PDFF maps on the level of L4 of a 39-year-old man (**A**) BMI: 24.92 kg/m^2^; mean PDFF abdominal muscles: 3.152%; mean PDFF psoas muscle: 0.764%; mean PDFF erector spinae: 6.332%; and a 37-year-old man (**B**) BMI: 30.68 kg/m^2^; mean PDFF abdominal muscles: 17.789%; mean PDFF psoas muscle: 12.678%; mean PDFF erector spinae: 11.386%.

**Figure 3:**
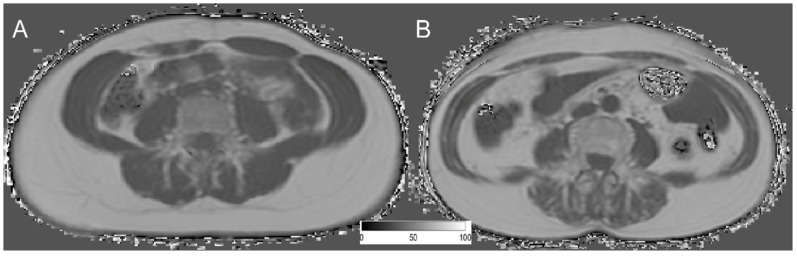
Representative PDFF maps on the level of L4 of a 23-year-old woman (**A**) BMI: 22.81 kg/m^2^; mean PDFF abdominal muscles: 1.569%; mean PDFF psoas muscle: 3.320%; mean PDFF erector spinae: 9.547%; and a 77-year-old woman (**B**) BMI: 23.39 kg/m^2^; mean PDFF abdominal muscles: 13.992%; mean PDFF psoas muscle: 3.162%; mean PDFF erector spinae: 29.698%.

The average PDFF of the erector spinae muscle was 7.99 ± 6.36% (range: 3.32–34.29%) in men and 14.87 ± 6.74% (range: 0.71–31.34%) in women (*p* = 0.011). No significant differences between men and women, regarding PDFF, could be detected in the psoas muscle (*p* = 0.650) and abdominal muscles (*p* = 0.262). However, all muscular compartments showed significant differences in CSA *(p* < 0.0001) (Table 1). Representative PDFF maps of the abdominal muscles, psoas muscle, and erector spinae muscles from men and women are shown in Figure 2 and Figure 3.

### 3.3. Correlations between Muscle PDFF, BMI, and Age

A positive correlation between BMI (*r* = 0.510, *p* = 0.022) and age (*r* = 0.638, *p* < 0.0001), with the abdominal muscle PDFF, could be detected in men (Table 2). However, partial correlation testing adjusting for BMI did not result in a significant correlation with age (*r* = 0.362, *p* = 0.128).

**Figure 4:**
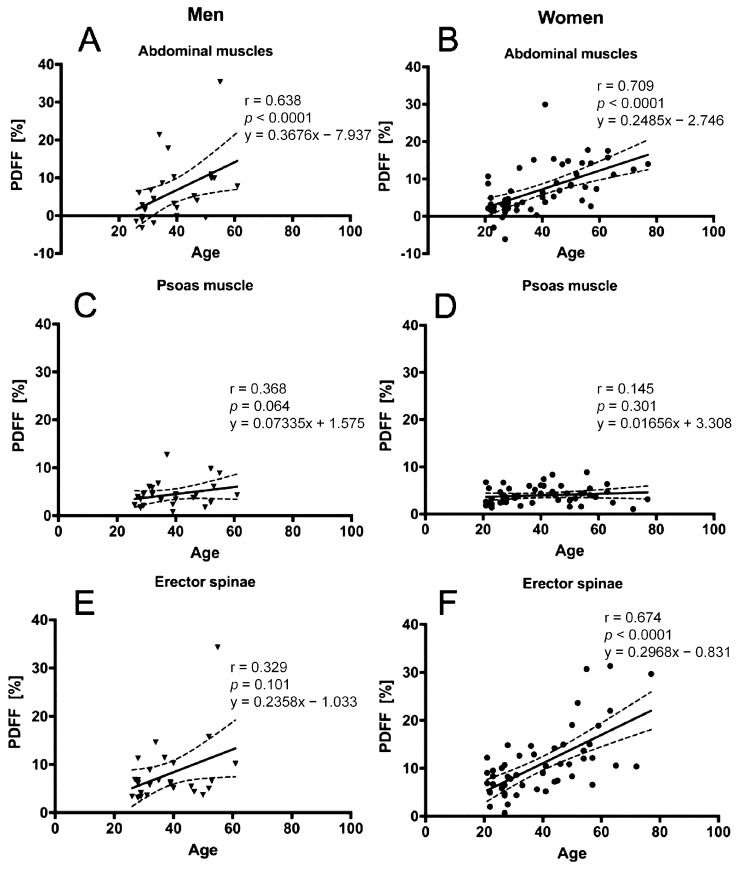
Proton density fat fraction (PDFF) of the abdominal muscle compartments. This figure plots the PDFF of the abdominal muscle, psoas muscle, and erector spinae against age for men (**A**,**C**,**E**) and women (**B**,**D**,**F**), respectively. The areas between the dotted lines represent the 95% confidence band of the best-fit line. Triangles represent x/y values for men and dots for women.

**Figure 5:**
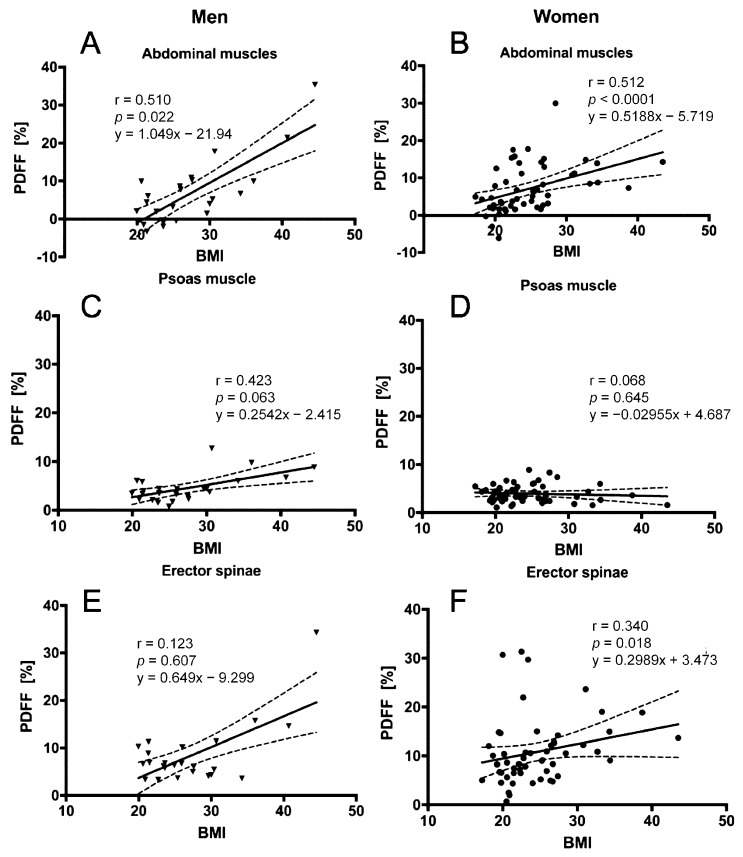
Proton density fat fraction (PDFF) of the abdominal muscle compartments. This figure plots the PDFF of the abdominal muscle, psoas muscle and erector spinae against BMI for men (**A**,**C**,**E**) and women (**B**,**D**,**F**), respectively. The areas between the dotted lines represent the 95% confidence band of the best-fit line. Triangles represent x/y values for men and dots for women.

In men and women, BMI (men: *r* = 0.510, *p* = 0.022; women: *r* = 0.512, *p* < 0.0001) and age (men: *r* = 0.638, *p* = 0.0001; women: *r* = 0.709, *p* < 0.0001) correlated significantly with the abdominal muscle PDFF. Additionally, correlations between BMI (*r* = 0.340, *p* = 0.018) and age (*r* = 0.674, *p* < 0.0001) with erector spinae muscle PDFF could be detected in women (Table 2 and Table 3). CSA of the erector spinae only showed significant correlation with BMI in men (*r* = 0.599, *p* = 0.005) (Table 4 and Table 5). PDFF of the different muscle compartments are plotted against age and BMI in Figure 4 and Figure 5.

When performing partial correlation testing with age as a control variable, statistical significance was preserved for BMI versus abdominal muscle PDFF in men and women (men: *r* = 0.510, *p* = 0.022; women: *r* = 0.308, *p* = 0.014) (Table 6 and Table 7).

When performing partial correlation testing with BMI as a control variable, statistical significance was only preserved for age versus abdominal muscle PDFF in women (*r* = 0.631, *p* < 0.0001) (Table 8).

## 4. Discussion

In this analysis, chemical shift encoding-based water–fat MRI was used to assess PDFF values of trunk muscles in healthy men and women. BMI turned out to have an influence on PDFF of the abdominal muscles in men, whereas, in women, statistically significant correlations were found for age and PDFF of the abdominal and erector spinae muscles. After consideration of BMI as a potential confounder variable, the correlation of abdominal muscle PDFF with age was preserved in women. Partial correlation analyses, in the context of this study, were performed to adjust for the influence of age and BMI, respectively. The present findings support the hypothesis that fat deposition in trunk muscles is driven primarily by age, rather than BMI, in women. In males, trunk muscle composition is BMI-associated, but it remains to be investigated whether the trunk muscle fat accumulation is also age-associated, as the relatively low number of male subjects has to be considered. Especially, the age group over 49 years is underrepresented in the male subjects, which could cause a bias of the presented results. An extended male cohort size might lead to similar associations with respect to age as in women.

There are several diseases which have an impact on muscle structure and composition, like type 2 diabetes, neuromuscular disorders, and lower back pain, amongst others [12,23,28,29,30]. Quantitative visualization of the compositional changes in muscle tissue accompanying these diseases strengthens our understanding of the interaction between the musculature and vertebral column [10,31,32]. Osteoporosis represents a disease entity with high prevalence and strong socioeconomic relevance through expansive costs, not only for fracture treatment, but also due to long-term medication expenses [33,34]. Various publications highlighted the changes in the paraspinal musculature with respect to fatty infiltration and reduced CSA. Such changes are associated with decreased bone mineral density and increased fracture risk in postmenopausal women [35]. In the present analysis, a non-significant inverse correlation between abdominal muscle CSA and age, and a highly significant correlation between abdominal muscle PDFF and age in women, could be observed. These findings underline the non-exclusively hormone-induced tendency of muscular volume reduction and fatty infiltration in women.

However, it has to be taken into consideration that not only in fully manifested diseases, like metabolic syndrome, can the described musculoskeletal alterations be observed. In subjects with a BMI considered to range from overweight to obese, increasing muscular PDFF values are detectable, and may indicate a pathological ectopic fat deposition. This information cannot be obtained from common anthropometric methods, and may serve as a complementary valuable parameter in the risk assessment of obesity and metabolic phenotypes. As a consequence, the presented technique of chemical shift encoding-based water–fat imaging for quantitative assessment of trunk muscle composition not only deepens our understanding of pathophysiological conditions present in musculature, but also offers the opportunity of clinical application in preventive medicine to better identify subjects at risk of metabolic diseases [2,36,37,38]. Our findings further foster the awareness that the described changes in muscular structure and composition not only vary according to the presence of specific diseases, but can also be detected in clinically healthy subjects. Against this background, a comparison of the muscular, subcutaneous, and visceral fat deposition, and their impact on metabolic health, would additionally be of interest [39].

The presented results have to be interpreted cautiously in certain aspects, because of methodic limitations. First, the relatively small cohort size has to be regarded as a main limitation. In particular, the absence of significant correlations in males could be related to the relatively low number of male subjects (*n* = 26). Studies with larger balanced cohorts are needed to further investigate the hypothesis that male muscular fat deposition is BMI- rather than age-associated. Second, this analysis focused on healthy subjects. Thus, more investigations are needed to analyze how specific pathological conditions affect trunk muscle PDFF in both sexes, and to explore the potential of PDFF values as biomarkers in clinical settings. Taking clinical information into account would help to put the information gained from this imaging technique into perspective. Third, the segmentation of one single slice of the acquired sequence is a further limiting factor. Partial volume effects caused by the relatively large voxel size have to be considered. Furthermore, the segmentation was not selectively performed on intramuscular fat. Hence, the PDFF of each muscle compartment calculated comprised both intra- and intermuscular fat.

## 5. Conclusions

In conclusion, the results of the present analysis suggest a primarily age-driven abdominal muscle fat accumulation in women, measured quantitatively with PDFF based on chemical shift encoding-based water–fat MRI. By contrast, in men, BMI seems to have a bigger impact on muscular fat deposition than age. However, the relatively low number of male subjects has to be kept in mind, and the results with respect to age-dependent ectopic fat deposition have to be interpreted cautiously. This study substantiates the potential of PDFF as a new quantitative imaging biomarker of ectopic fat in muscle, but larger studies with more intensive metabolic phenotyping are needed for further confirmation.

## Figures and Tables

**Table 1:** Subject characteristics (age and body mass index (BMI)), PDFF values, and cross-sectional area (CSA) of abdominal muscles, psoas muscle, and erector spinae. Parameters were compared between the two groups with Mann–Whitney tests (*p*-values).

	Subjects	*n*	Mean	SD	*p*
age	men	26	38.85	10.38	0.665
(years)	women	53	39.51	15.03	
BMI	men	20	26.37	5.30	0.455
(kg/m^2^)	women	48	25.67	5.46	
PDFF_abdominal muscles_	men	26	6.16	8.26	0.262
(%)	women	53	6.96	6.52	
CSA_abdominal muscles_	men	26	43.83	9.87	<0.0001
(a.u.)	women	53	27.78	6.56	
PDFF_psoas muscle_	men	26	4.45	2.76	0.650
(%)	women	53	3.93	1.79	
CSA_psoas muscle_	men	26	17.88	4.11	<0.0001
(a.u.)	women	53	10.81	2.48	
PDFF_erector spinae_	men	26	7.99	6.36	0.011
(%)	women	53	14.87	31.74	
CSA_erector spinae_	men	26	37.40	6.77	<0.0001
(a.u.)	women	53	25.02	4.74	

a.u. means arbitrary units.

**Table 2:** This table illustrates the results of the correlation of age, body mass index (BMI), and proton density fat fraction (PDFF) in the abdominal muscles, psoas muscle, and erector spinae in men. Spearman’s rho correlation coefficients are provided for each correlation, and *p*-values indicate statistical significance of the respective correlation (n.s.: not significant).

		Age	BMI	PDFF_abdominal muscles_	PDFF_psoas muscle_	PDFF_erector spinae_
age	Spearman’s rho	1		0.638		
(years)	*p*	-	n.s.	0.0001	n.s.	n.s.
BMI	Spearman’s rho		1	0.510		
(kg/m^2^)	*p*	n.s.	-	0.022	n.s.	n.s.
PDFF_abdominal muscles_	Spearman’s rho	0.638	0.510	1	0.543	0.395
(%)	*p*	0.0001	0.022	-	0.004	0.046
PDFF_psoas muscle_	Spearman’s rho			0.543	1	0.506
(%)	*p*	n.s.	n.s.	0.004	-	0.008
PDFF_erector spinae_	Spearman’s rho			0.395	0.506	1
(%)	*p*	n.s.	n.s.	0.046	0.008	-

**Table 3:** In this table Spearman’s rho correlation is depicted for age, BMI, and PDFF in the different muscle compartments in women.

		Age	BMI	PDFF_abdominal muscles_	PDFF_psoas muscle_	PDFF_erector spinae_
age	Spearman’s rho	1	0.324	0.709		0.674
(years)	*p*	-	0.025	0.0001	n.s.	0.0001
BMI	Spearman’s rho	0.324	1	0.512		0.340
(kg/m^2^)	*p*	0.025	-	0.0001	n.s.	0.018
PDFF_abdominal muscles_	Spearman’s rho	0.709	0.512	1		0.653
(%)	*p*	0.0001	0.001	-	n.s.	0.0001
PDFF_psoas muscle_	Spearman’s rho				1	
(%)	*p*	n.s.	n.s.	n.s.	-	n.s.
PDFF_erector spinae_	Spearman’s rho	0.674	0.340	0.653		1
(%)	*p*	0.0001	0.018	0.0001	n.s.	-

**Table 4:** This table illustrates the results of the correlation of age, body mass index (BMI), and cross-sectional area (CSA) in the abdominal muscles, psoas muscle, and erector spinae in men.

		Age	BMI	CSA_abdominal muscles_	CSA_psoas muscle_	CSA_erector spinae_
age	Spearman’s rho	1				
(years)	*p*	-	n.s.	n.s.	n.s.	n.s.
BMI	Spearman’s rho		1			0.599
(kg/m^2^)	*p*	n.s.	-	n.s.	n.s.	0.005
CSA_abdominal muscles_	Spearman’s rho			1		
(a.u.)	*p*	n.s.	n.s.	-	n.s.	n.s.
CSA_psoas muscle_	Spearman’s rho				1	
(a.u.)	*p*	n.s.	n.s.	n.s.	-	n.s.
CSA_erector spinae_	Spearman’s rho		0.599			1
(a.u.)	*p*	n.s.	0.005	n.s.	n.s.	-

**Table 5:** This table illustrates the results of the correlation of age, body mass index (BMI), and cross-sectional area (CSA) in the abdominal muscles, psoas muscle, and erector spinae in women.

		Age	BMI	CSA_abdominal muscles_	CSA_psoas muscle_	CSA_erector spinae_
age	Spearman’s rho	1	0.324			
(years)	*p*	-	0.025	n.s.	n.s.	n.s.
BMI	Spearman’s rho	0.324	1			
(kg/m^2^)	*p*	0.025	-	n.s.	n.s.	n.s.
CSA_abdominal muscles_	Spearman’s rho			1	0.558	0.424
(a.u.)	*p*	n.s.	n.s.	-	0.0001	0.002
CSA_psoas muscle_	Spearman’s rho			0.558	1	
(a.u.)	*p*	n.s.	n.s.	0.0001	-	n.s.
CSA_erector spinae_	Spearman’s rho			0.424		1
(a.u.)	*p*	n.s.	n.s.	0.002	n.s.	-

**Table 6:** Partial correlations for men with age as a control variable.

		BMI	PDFF_abdominal muscles_	PDFF_psoas muscle_	PDFF_erector spinae_
BMI	*r*	1	0.555		
(kg/m^2^)	*p*	-	0.014	n.s.	n.s.
PDFF_abdominal muscles_	*r*	0.510	1	0.618	0.555
(%)	*p*	0.022	-	0.005	0.014
PDFF_psoas muscle_	*r*		0.618	1	0.620
(%)	*p*	n.s.	0.005	-	0.005
PDFF_erector spinae_	*r*		0.464	0.620	1
(%)	*p*	n.s.	0.045	0.005	-

**Table 7:** Partial correlations for women with age as a control variable.

		BMI	PDFF_abdominal muscles_	PDFF_psoas muscle_	PDFF_erector spinae_
BMI	*r*	1	0.308		
(kg/m^2^)	*p*	-	0.014	n.s.	n.s.
PDFF_abdominal muscles_	*r*	0.308	1		
(%)	*p*	0.014	-	n.s.	n.s.
PDFF_psoas muscle_	*r*			1	
(%)	*p*	n.s.	n.s	-	n.s.
PDFF_erector spinae_	*r*				1
(%)	*p*	n.s.	n.s.	n.s.	-

**Table 8:** Partial correlations for women with BMI as a control variable.

		Age	PDFF_abdominal muscles_	PDFF_psoas muscle_	PDFF_erector spinae_
age	*r*	1	0.631		
(kg/m^2^)	*p*	-	<0.0001	n.s.	n.s.
PDFF_abdominal muscles_	*r*	0.631	1	0.293	
(%)	*p*	<0.0001	-	0.046	n.s.
PDFF_psoas muscle_	*r*		0.293	1	0.538
(%)	*p*	n.s.	0.046	-	0.017
PDFF_erector spinae_	*r*				1
(%)	*p*	n.s.	n.s.	n.s.	-
